# No training required: experimental tests support homology-based DNA assembly as a best practice in synthetic biology

**DOI:** 10.1186/s13036-015-0006-z

**Published:** 2015-06-12

**Authors:** Afnan Azizi, Wilson Lam, Hilary Phenix, Lioudmila Tepliakova, Ian J Roney, Daniel Jedrysiak, Alex Power, Vaibhav Gupta, Nada Elnour, Martin Hanzel, Alexandra C Tzahristos, Shihab Sarwar, Mads Kærn

**Affiliations:** Ottawa Institute of Systems Biology, 451 Smyth Road, K1H 8M5 Ottawa, Ontario Canada; Department of Cellular and Molecular Medicine, University of Ottawa, 451 Smyth Road, K1H 8M5 Ottawa, Ontario Canada; Biochemistry Program, University of Ottawa, Gendron Hall, K1N 6N5 Ontario, Canada; Biomedical Sciences Program, University of Ottawa, Marion Hall, K1N 6N5 Ottawa, Ontario Canada; Department of Physics, University of Ottawa, MacDonald Hall, K1N 6N5 Ottawa, Ontario Canada

**Keywords:** Synthetic biology, DNA assembly, Cloning, Gibson, Seamless, iGEM, PCR

## Abstract

The Registry of Standard Biological Parts imposes sequence constraints to enable DNA assembly using restriction enzymes. Alnahhas et al. (Journal of Biological Engineering 2014, 8:28) recently argued that these constraints should be revised because they impose an unnecessary burden on contributors that use homology-based assembly. To add to this debate, we tested four different homology-based methods, and found that students using these methods on their first attempt have a high probability of success. Because of their ease of use and high success rates, we believe that homology-based assembly is a best practice of Synthetic Biology, and recommend that the Registry implement the changes proposed by Alnahhas et al. to better support their use.

**Electronic supplementary material:**

The online version of this article (doi:10.1186/s13036-015-0006-z) contains supplementary material, which is available to authorized users.

The Registry of Standard Biological Parts (the Registry) is a repository of DNA material that aims to make biology easier to engineer by facilitating the sharing of genetic parts and simplifying their assembly. The Registry also sets the rules and standards for the International Genetically Engineered Machine (iGEM) competition, and is in this capacity highly influential on the formation of the next generation of bioengineers and biotechnology entrepreneurs. The Registry currently recommends the use of a DNA assembly method called three-antibiotic (3A) restriction enzyme assembly [1], and genetic parts submitted to the repository must comply with sequence restrictions that are necessary for this method to work. Although the Registry mention homology-based assembly on their website, 3A assembly is the only recommended method.

Alnahhas et al. [2] recently advocated that the Registry should no longer enforce compatibility with 3A assembly because homology-based assembly methods are highly versatile and efficient, and free of the sequence constraints imposed by the use of restriction enzymes. The authors argue that the cost and time needed to eliminate all illegal sequences imposes a significant and unnecessary burden on contributors, and provide survey data indicating that many iGEM teams have already adopted homology-based assembly. They recommend a new submission standard that would significantly reduce the occurrence of illegal sequences while retaining the quality control features of the current submission standard.

Should the Registry recommend homology-based assembly along with 3A assembly and abandon one of its founding principles? Our lab did not have extensive experience with homology-based methods, so to inform ourselves about their benefits and limitations, we tested the performance of four different methods (Fig. 1). We specifically examined how well each method facilitated the assembly of a circularized plasmid in yeast *Saccharomyces cerevisiae* that naturally is able to fuse overlapping DNA fragments during a transformation without specialized enzymes or reagents [3]. This *in vivo* homologous recombination (HR) method served as our baseline.

**Fig. 1:**
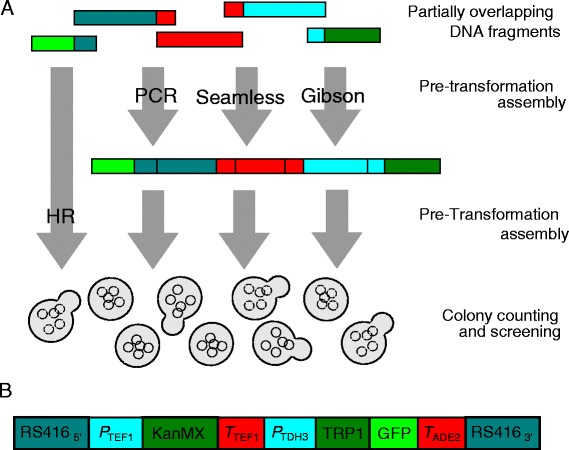
Overview. **a** Partially overlapping DNA fragments are transformed into yeast cells together with a linearized plasmid backbone, or fused together by PCR, Seamless or Gibson assembly prior to the transformation. Homologous recombination (HR) enables the fusion of the DNA fragments without transformation. **b** The assembled plasmid insert contains 4.5 kb DNA encoding two expression units, the *TEF1* promoter driving *KanR* expression, and the *TDH3* promoter driving a fusion of the *TRP1* and *GFP* genes. The insert has DNA sequences at the ends that are homologous to the ends of the linearized plasmid backbone (not shown). Assembly success was tested when the insert was broken into two, three, four or five fragments with short or long regions of homology to neighboring fragments or the linearized RS416 plasmid. The transformation used 2 ng or 20 ng of total DNA, including the linearized plasmid. The linearized plasmid DNA was added at the pre-transformation step for Seamless and Gibson assembly

We compared HR assembly to three *in vitro* methods where DNA fragments are fused prior to transformation: PCR assembly based on overlap extension [4, 5], Seamless assembly [6] and Gibson assembly [7]. Because HR takes place in all our tests, we expected that adding *in vitro* assembly prior to the transformation would increase the probability of cloning success. We did not have the opportunity to test Ligase-cycling assembly [8, 9], nor commercially available PCR-based methods. Neither did we test 3A assembly, and can therefore not compare it directly to homology-based assembly. It is worth noting, however, that 3A assembly only allows for the fusion of two DNA fragments and must be used reiteratively in assemblies requiring the fusion of more than two DNA fragments, which includes most of the assemblies tested in our experiments.

Our laboratory mostly uses DNA assembly for the construction of relatively small expression cassettes that report on cellular signaling pathways or form building blocks of larger gene regulatory networks. To test the utility of homology-based assembly in this context, we decided to construct a circular plasmid with a 4.5 kb insert that encodes two expression cassettes (Fig. 1b): a cassette where the *KanR* gene, conferring resistance to G418, is expressed from the promoter of the *TEF1* gene, and a cassette where a fusion of the *TRP1* and *GFP* genes is expressed from the promoter of the *TDH3* gene. The 5’ and 3’ ends of the assembled insert contain sequence homologies to a shuttle vector backbone (pRS416). This commonly used vector has origins of replication for *E. coli* and yeast, as well as selection cassettes for *E. coli* (*AmpR* confers resistance to ampicillin) and yeast (*URA3MX* allows cells that are auxotrophic for uracil to grow in the absence of uracil) [10].

To cover a broad range of conditions, we performed 192 assembly experiments that tested each of the four methods under 16 different conditions. In these conditions, the vector backbone was unchanged while the insert was assembled from two, three, four or five DNA fragments that had either long (200 bp or more) or short (~30-40 bp) homologous regions to one another. We tested such short “overhangs” because they can be added to low-cost PCR primers, but note that long overhangs can be created at a relatively low cost if overlap extension PCR is used to fuse two or more parts prior to the assembly. We then transformed yeast cells with either a low (2 ng) or high (20 ng) total DNA.

We generally expect that method developments reported in the peer-reviewed literature involve personnel with considerable experience and practice with its application. To assess if the four assembly methods are efficient also when used by personnel without such experience, we had the assembly reactions and subsequent transformations done independently by an MSc student (A.A. with assistance from V.G.), a PhD student (H.P.) and a laboratory technician (L.T.). Each person was allowed to perform each test only once. While L.T. did a few preliminary experiments, the results reported for A.A. and H.P. were obtained when they used our assembly protocols for the first time (the protocols are provided with this letter [Additional file 1]).

For each of the 192 tests, the plasmid assembly was judged successful if the transformation allowed a colony 1) to grow in the absence of uracil, 2) to fluoresce green light and 3) to be resistant to G418. For each test, we either screened 20 colonies or all colonies when 20 colonies were not available (17 % of the tests). Remarkably, we observed a 96 % overall success with only seven of the 192 tests failing to yield at least one colony with a fully functional plasmid. The more experienced performers (H.P. and L.T.) were successful in all of their 128 tests. Moreover, at least one positive colony was found in 95 of 96 tests with 20 ng transformed DNA, and in all the tests that used Gibson assembly irrespective of conditions. The method that failed most often was PCR assembly (five failures in 48 tests). Some of these failures likely arise from human error during repetitive operations (e.g., missing template DNA or primers).

While the four methods worked remarkably well, the screening of up to 20 colonies per assembly can be both expensive and impractical. For this reason, we assessed how well the methods work when fewer colonies are screened. To do this, we calculated for each test the probability that screening three colonies would give at least one colony with a fully functional plasmid, and judged a test as successful if this probability was 95 % or higher. We then counted for each method the fraction of successful tests, and used these fractions to calculate overall success rates as well as to compare the four methods. With this more restrictive measure of assembly success, we found that 64 % of the 192 tests were successful (Fig. 2a). While HR alone was successful in 44 % of the tests that used this method, the tests that used PCR, Seamless or Gibson assembly had success rates of 56 %, 73 % or 81 %, respectively.

**Fig. 2:**
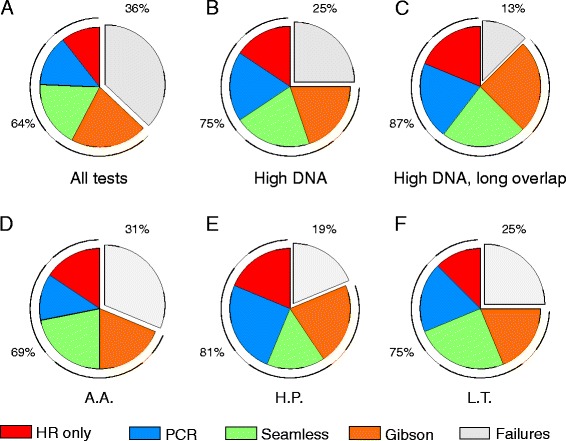
Charts illustrating the overall success rate and the success rates for each assembly method under different conditions. Success is defined as a 95 % hypergeometric probability or higher that at least one of three clones screened carry a fully functional plasmid. The fraction of failed tests is indicated in grey. The fraction of successful tests is subdivided into different colors to indicate the method used. **a** All tests. Overall success rate: 65 %. Individual method success rates: 44/56/73/81 % for HR alone, PCR, Seamless and Gibson, respectively. **b** Tests with 20 ng transformed DNA. Overall: 75 %. Methods: 65/75/83/79 %. **c** Tests with 20 ng transformed DNA and long regions of overlap between DNA fragments. Overall: 87 %. Methods: 75/83/92/100 %. **d** Tests performed by A.A. Overall: 69 %. Methods: 61/50/88/75 %. **e** Tests performed by H.P. Overall: 81 %. Methods: 75/100/62/88 %. **f** Tests performed by L.T. Overall: 75 %. Methods: 50 %/75 %/100 %/75 %

Several conditions resulted in increased success rates. Notably, 75 % of the 96 tests that used 20 ng total DNA were successful. The success rates for HR alone, PCR and Seamless assembly were in these tests 63 %, 75 % and 83 %, respectively (Fig. 2b). Gibson assembly was insensitive to the amount of transformed DNA, presumably because the concentration of correctly assembled plasmid is higher with this method than with the others. This would be consistent with the observation that Gibson assembly always yielded many more colonies than any of the other methods (data not shown). Interestingly, we did not see a consistent decrease in success rate with an increase in the number of fragments. In tests with 20 ng transformed DNA, the assembly of three fragments failed in nine of 24 tests while the assembly of four fragments failed only four times in 24 tests. This could reflect a sensitivity of homology-based assembly to the sequence of the overlap of the DNA fragments.

High success rates were obtained in tests with 20 ng transformed DNA and long regions of homology (Fig. 2c). In these cases, 87 % of 48 tests were successful with success rates of 75 %, 83 %, 92 % and 100 % for HR alone, PCR, Seamless and Gibson assembly, respectively. The success rates were also high in tests that used two DNA fragments and 20 ng transformed DNA. In the 24 tests done under these conditions, we found that 23 of them had a 95 % probability or higher that at least one colony has a fully functional plasmid. Remarkably, this probability was 99 % or higher, irrespective of the assembly method and the length of the homologous overhangs, in the 16 tests that used two DNA fragments and 20 ng transformed DNA and completed by H.P. or L.T. In fact, for the more experienced personnel, almost all tests with two DNA fragments and 20 ng transformed DNA would have been successful even if only one colony had been examined.

To further evaluate the impact of individual training level, we examined the percentage of successful assembly tests for each of the three performers (Fig. 2d-f). As one might anticipate, the performer with the least experience also had a lower overall success rate (69 % for A.A. compared to 81 % for H.P. and 75 % for L.T.). Interestingly, the success rate for each method varied considerably among the performers. For example, while H.P. attained a 100 % success rate with PCR assembly, Seamless assembly was the only method where the most experienced performer (L.T.) had a perfect record. All of the performers achieved a 100 % success rate in the tests of Gibson assembly, but only in tests where long regions of homology were used. Correspondingly, none of the assembly methods stands out as consistently better compared to the others across all conditions. However, compared to HR alone, performing *in vitro* assembly prior to transformation using the PCR, Seamless or Gibson assembly did increase the probability of cloning success under all conditions, and especially so when a low amount of DNA was used in the transformation step.

Our data demonstrates that personnel with no specialized training in the first attempt have a high likelihood of successfully using homology-based assembly. For example, for the one-step insertion of two DNA fragments into a plasmid backbone, the cloning was successful in 23 of 24 of the tests that used 20 ng transformed DNA. However, the most important benefit of homology-based assembly is the ability to fuse more than two DNA fragments in a single step. Notably, in our tests fusing four DNA fragments, the cloning was successful in 20 of 24 experiments with 20 ng transformed DNA. For comparison, conventional binary restriction enzyme cloning would require a minimum of three steps and likely involve the screening of at least six colonies. In our tests, screening six colonies would give a 99 % probability of success in 23 of the 24 experiments that used four DNA fragments and 20 ng transformed DNA.

Our results have convinced us that homology-based assembly for our purposes provide significant benefits. For this reason, we agree with Alnahhas et al. that the Registry should no longer enforce compatibility with 3A assembly. We also hope that our results will encourage teams participating in the iGEM competition to test if homology-based assembly might be beneficial to them. Because our data do not allow a direct cost-benefit comparison to 3A assembly, it would be valuable if teams tested the efficiency, accuracy and cost of both 3A assembly and homology-based assembly. In fact, we are unaware of any systematic tests documenting the efficiency and accuracy of 3A assembly by inexperienced and unassisted personnel, and believe that such tests are critical to ensure that the results reflect what would be achievable by most iGEM participants.

We recognize that homology-based assembly methods have shortcomings. As pointed out to us by Tom Knight (personal communication), major issues are that failed PCR reactions are difficult to troubleshoot, and are likely to perform poorly for assemblies that involve identical or near-identical parts. Other drawbacks are the need for assembly-specific primers, which may not be readily available in some areas (i.e., developing countries), and that a cleanup may be required to eliminate off-target fragments from the initial amplification of overlapping DNA fragments.

Our results demonstrate that the shortcomings of homology-based methods for our purposes are relatively minor compared to the increase in productivity they provide. The assembly protocols we used gave high success rates across a broad range of conditions, did not require troubleshooting nor prior practice, and were time efficient. Colonies could in most cases be screened two days after the *in vitro* assembly and each performer was able to successfully complete roughly 20 assemblies per week. Whether homology-based assembly will be equally useful to others will require further testing. Specifically, the commercial Seamless and Gibson assembly kits are relatively expensive and may not always be cost-efficient because *in vivo* recombination-based methods have a very low cost per reaction. Correspondingly, we believe it would benefit the Synthetic Biology community to further develop and test such methods, especially for assembly in *E. coli* [11].

In conclusion, our results have convinced us that homology-based assembly for our purposes is a best practice of Synthetic Biology. Because of this, we believe the Registry should implement the suggestions by Alnahhas et al. and recommend the use of homology-based assembly along with 3A assembly. In the light that many iGEM teams already use homology-based assembly, we believe that these steps are vital to maintain the relevance of the Registry as a community resource, and the usefulness of the iGEM competition as a vehicle for Synthetic Biology training. While it is noble that the Registry seeks to maintain a level playing field for participants in the iGEM competition, it might not be sustainable to achieve this objective by impeding the use of technology advancements.

## Additional file

Additional file 1: Methods and protocols for “No training required: Experimental tests support homology-based DNA assembly as a best practice in Synthetic Biology”.
