# Erratum to: No training required: experimental tests support homology-based DNA assembly as a best practice in synthetic biology

**DOI:** 10.1186/s13036-015-0013-0

**Published:** 2016-01-07

**Authors:** Afnan Azizi, Wilson Lam, Hilary Phenix, Lioudmila Tepliakova, Ian J. Roney, Daniel Jedrysiak, Alex Power, Vaibhav Gupta, Nada Elnour, Martin Hanzel, Alexandra C. Tzahristos, Shihab Sarwar, Mads Kærn

**Affiliations:** Ottawa Institute of Systems Biology, 451 Smyth Road, K1H 8 M5 Ottawa, ON Canada; Department of Cellular and Molecular Medicine, University of Ottawa, 451 Smyth Road, K1H 8 M5 Ottawa, ON Canada; Biochemistry Program, University of Ottawa, Gendron Hall, K1N 6 N5 Ottawa, ON Canada; Biomedical Sciences Program, University of Ottawa, Marion Hall, K1N 6 N5 Ottawa, ON Canada; Department of Physics, University of Ottawa, MacDonald Hall, K1N 6 N5 Ottawa, ON Canada

## Erratum

After the publication of this work [1], we noticed that an incorrect version of Fig. 1 was published. The captions for Figs. 1 and 2 were also incorrect. The correct version of Fig. 1 and the captions for Figs. 1 and 2 are provided below. The publisher apologises for any inconvenience caused.

**Fig. 1:**
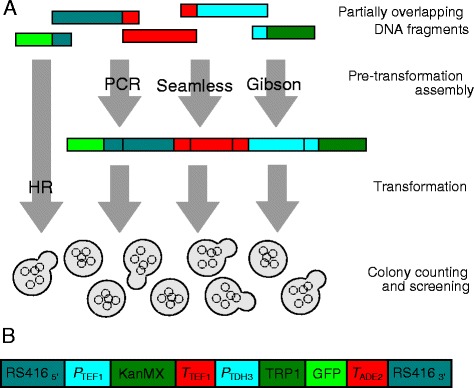
Overview. **a** Partially overlapping DNA fragments are transformed into yeast cells together with a linearized plasmid backbone, or fused together by PCR, Seamless or Gibson assembly prior to the transformation. Homologous recombination (HR) enables the fusion of the DNA fragments without pretransformation assembly. **b** The assembled plasmid insert contains 4.5 kb DNA encoding two expression units, the TEF1 promoter driving KanR expression, and the TDH3 promoter driving a fusion of the TRP1 and GFP genes. The insert has DNA sequences at the ends that are homologous to the ends of the linearized plasmid backbone (not shown). Assembly success was tested when the insert was broken into two, three, four or five fragments with short or long regions of homology to neighboring fragments or the linearized RS416 plasmid. The transformation used 2 ng or 20 ng of total DNA, including the linearized plasmid. The linearized plasmid DNA was added at the pre-transformation step for Seamless and Gibson assembly

**Fig. 2:**
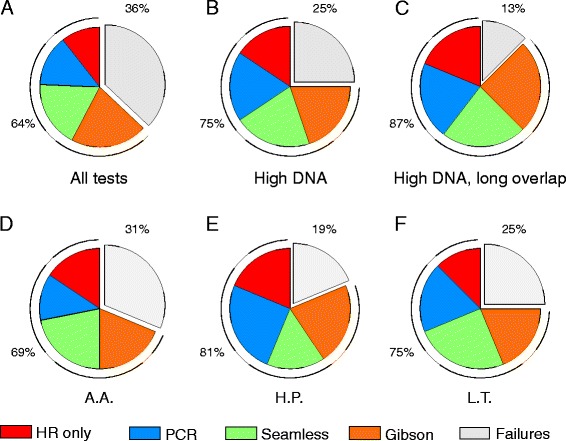
Charts illustrating the overall success rate and the success rates for each assembly method under different conditions. Success is defined as a 95 % hypergeometric probability or higher that at least one of three clones screened carry a fully functional plasmid. The fraction of failed tests is indicated in grey. The fraction of successful tests is subdivided into different colors to indicate the method used. **a** All tests. Overall success rate: 65 %. Individual method success rates: 44/56/73/81 % for HR alone, PCR, Seamless and Gibson, respectively. **b** Tests with 20 ng transformed DNA. Overall: 75 %. Methods: 63/75/83/79 %. **c** Tests with 20 ng transformed DNA and long regions of overlap between DNA fragments. Overall: 87 %. Methods: 75/83/ 92/100 %. **d** Tests performed by A.A. Overall: 69 %. Methods: 61/50/88/75 %. **e** Tests performed by H.P. Overall: 81 %. Methods: 75/100/62/88 %. **f** Tests performed by L.T. Overall: 75 %. Methods: 50 %/75 %/100 %/75 %
