# Supplementary material for: No training required: experimental tests support homology-based DNA assembly as a best practice in synthetic biology
Source: J Biol Eng. 2015 Jun 12;9:8. doi: 10.1186/s13036-015-0006-z (PMC4464999; doi:10.1186/s13036-015-0006-z)
Supplement: Additional file 1: — Methods and protocols for “No training required: Experimental tests support homology-based DNA assembly as a best practice in Synthetic Biology”. [file 13036_2015_6_MOESM1_ESM.pdf]

Methods and protocols for "No training required: Experimental tests support homology-based DNA assembly as a best practice in Synthetic Biology"

Afnan Azizi[1,2]*, Wilson Lam[1,3]*, Hilary Phenix[1,2]*, Lioudmila Tepliakova[1,2]*, Ian J. Roney[1,2], Daniel Jedrysiak[1,2], Alex Power[1,2], Vaibhav Gupta[1,2], Nada Elnour[1,2], Martin Hanzel[1,4], Alexandra C. Tzahristos[1,3], Shihab Sarwar[1,3] and Mads Kærn[1,2,5]

1 Ottawa Institute of Systems Biology. 451 Smyth Road. Ottawa. Ontario. Canada K1H 8M5
2 Department of Cellular and Molecular Medicine, University of Ottawa. 451 Smyth Road. Ottawa. Ontario. Canada K1H 8M5
3 Biochemistry Program, University of Ottawa. Gendron Hall. Ontario. Canada. K1N 6N5
4 Biomedical Sciences Program, University of Ottawa. Marion Hall. Ottawa. Ontario. Canada K1N 6N5
5 Department of Physics, University of Ottawa. MacDonald Hall. Ottawa. Ontario. Canada K1N 6N5
*These authors contributed equally to this work. Correspondence should be addressed to M.K (mkaern@uottawa.ca)

DNA preparation 2
Homologous recombination assembly 3
Seamless assembly 4
Gibson assembly 5
PCR assembly 6
Yeast transformation 8
References 13

DNA preparation

Before beginning the series of assemblies, we used PCR amplification to prepare concentrated stock solutions of all DNA fragments including linearized vector, such that the same DNA stocks could be used for all methods and conditions. To obtain high enough concentrations of DNA stocks, we combined multiple samples as described below. Stocks were stored at -20 °C.

DNA fragments: Each fragment was amplified by PCR in ten separate reactions from the same master mix. DNA was purified (protocol below) for each reaction individually, and then pooled to obtain a final volume of ~10 x 30 µl in a 1.5 ml microcentrifuge tube. We evaporated the liquid in each tube by placing it in a 37 °C incubator with open cap until complete evaporation (~2 days), and resuspended the precipitated DNA with 30 µl of filter-sterilized ddH2O. We used the same fragment stocks for all assembly methods.

Vector: We linearized pRS416 by digesting it with SacI at 37 °C for 16 h, followed by enzyme deactivation at 65 °C for 25 min. We confirmed digestion by gel electrophoresis, purified DNA from the digestion reaction (protocol below), and then amplified and concentrated the linearized vector as described for the DNA fragments. We note that amplification excluded 111 bp of original pRS416, allowing it to have the correct flanking homologies to the assembled fragments. We used two stocks of vector for all assembly methods.

Homologous recombination assembly

Directly transform yeast with a DNA solution, containing vector and fragments of interest prepared as in the DNA preparation section. The solution should contain *x/2* ng of vector DNA and *x/(2*y)* ng of each non-vector fragment, where *x* is the total DNA mass used (2 ng or 20 ng) for the transformation and *y* is the total number of DNA fragments excluding the vector. Perform appropriate dilutions with filter-sterilized ddH_2O and keep on ice prior to transformation.

Seamless assembly

We used the GeneArt® Seamless Cloning and Assembly Enzyme Mix (Life Technologies, Catalogue No. A14606) and the following protocol modified by Afnan Azizi and Wilson Lam from [1]:

1. In a microcentrifuge tube, set up the seamless cloning and assembly reaction. **It is crucial that you add the GeneArt® 2X Enzyme Mix as the last component** (see Step 2).

DNA fragment(s) (200 ng each)	*x* µl
Linearized vector (200 ng)	*y* µl
Filter-sterilized ddH_2O	to 5 µl (none when [*x* + *y*] exceeds 5 µl)

2. Quickly thaw the GeneArt® 2X Enzyme Mix on ice and pipette up and down to mix thoroughly. Add 5 µl (or [*x* + *y*] when exceeding 5 µl) of the thawed GeneArt® 2X Enzyme Mix to each reaction mixture. Immediately return Enzyme Mix to -20 °C if it will be re-used within 1 day, otherwise return to -80 °C.

3. Mix the reaction components completely by pipetting them up and down.

4. Incubate at room temperature for 25 min, then keep in -20 °C until transformation.

5. For the subsequent transformation, calculate the volume (*x*) of reaction mix needed as: (*x* µl) = (*y* ng) / (*z* ng/µl), where *y* is the desired total DNA mass used for transformation and *z* is the concentration of total DNA in reaction mix based on DNA mass added before assembly. Perform all dilutions with filter-sterilized ddH_2O and keep on ice.

Gibson assembly

We used the Gibson Assembly® Cloning Kit (New England Biolabs Catalogue no. E5510S) and the following protocol modified by Afnan Azizi and Wilson Lam from [2]:

1. Use the equation pmols = (mass in ng) x 1,000 / (base pairs x 650 g/mol) to calculate the mass of each vector and non-vector DNA fragment needed to obtain the desired pmols described below.

2. Set up the following reaction on ice:

Non-vector fragments (γ * pmol/fragment)	*x* µl
Linearized vector (0.02 pmol)	*y* µl
2x Gibson Assembly Master Mix	10 µl
Filter-sterilized ddH_2O	to 20 µl

   * For up to 2 fragments $\gamma = 0.02$, otherwise $\gamma = 0.04$.

3. Incubate samples at 50 °C for 60 min. Following incubation, store samples on ice or at -20° C for subsequent transformation.

4. For subsequent transformation, calculate volume (*x*) of reaction mix needed as: (*x* µl) = (*y* ng) / (*z* ng/µl), where *y* is the desired total DNA mass used for transformation and *z* is the concentration of total DNA in the reaction mix based on DNA mass added before assembly. Perform all dilutions with filter-sterilized ddH_2O and keep on ice.

PCR assembly

We used the following protocol adapted by Alexander Power and Daniel Jedrysiak based on methods described in [3] and [4]:

Reaction Mix

Prepare the following on ice in PCR tubes, mix by pipetting. **Do not add primers until reaction mix has been thermocycled using PCR Program Step 1 to 6.**

50ng/1000bp of each DNA fragment	*x* µl
5x Phusion HF Reaction Buffer (NEB #B0518S)	5 µl
Oligonucleotide primer pair (10 pmol/µl each)	2 * 1.25 µl
dNTP Solution Mix (NEB #N0447S)	0.5 µl
2 U/µl Phusion DNA Polymerase (Thermo Scientific F-530L)	0.5 µl
Filter-sterilized ddH_2O	up to 25 µl

PCR Program

In this study, the following program was used for all conditions:

1. 98 °C 3 min
2. 98 °C 10 s
3. *X* °C 25 s
4. 72 °C 105 s
5. Repeat Steps 2-4 for a total of 10 cycles
6. Pause (Remove tube from PCR machine and add primer pair, mix by pipetting).
7. 98 °C 10 s
8. *X* °C 25 s
9. 72 °C 105 s
10. Repeat steps 7-9 for a total of 25 cycles
11. 72 °C 10 min
12. 4 °C 4 min

X = annealing temperature based on primer design.

The same terminal primer pair was used for all reactions involving long overhangs (*X* = 59). A second terminal primer pair was used for those with short overhangs (*X* = 55).

Preparation of DNA for transformation

Following PCR, verify assembly by running 2 µl of PCR reaction mix using standard gel procedure. Purify DNA (protocol below) from the remaining reaction mix and re-measure DNA concentration.

For subsequent transformation, use purified PCR DNA directly and dilute in filter-sterilized ddH_2O if necessary and keep on ice. Otherwise store at -20 °C.

Note: In this study, we used *x*/2 ng of purified PCR DNA and *x*/2 ng of linearized vector DNA (preparation described above) for subsequent transformation, where *x* is the desired total mass of DNA to be used for transformation.

Spin Column PCR Product Purification

We used the EZ-10 Spin Column PCR Products Purification Kit (Bio Basic Inc., Catalogue no. BS664) and the following protocol adapted by Mila Tepliakova from [5]:

1. Relative to volume of PCR reaction, add 3 volumes of Binding Buffer I directly to PCR reaction tube and mix by pipetting up and down.
2. Transfer the above solution to the EZ-10 column and let it stand at room temperature for 2 min. Centrifuge at 9300 *x g* for 2 min.
3. Remove the flow-through in the tube. Add 750 µl of Wash Solution to the column and centrifuge at 9300 *x g* for 2 min.
4. Remove the flow-through in the tube and centrifuge at 9300 *x g* for 3 min to remove residual Wash Solution.
5. Transfer the column to a clean 1.5 ml microcentrifuge tube. With cap open, keep at room temperature for 1h to allow residual Wash Solution to evaporate.
6. Using a micropipette, dispense 30 µl of filter-sterilized ddH_2O onto the centre of the column. Incubate at room temperature for 30 min. Centrifuge at 9300 *x g* for 2 min to elute the DNA. Note that it is extremely important to add ddH_2O to the centre part of the column.

Yeast transformation

We used the following protocol adapted by Mila Tepliakova from [6]:

Preliminary notes

This protocol yields up to 10 transformation reactions including controls.

Multiple steps within this protocol indicate application of “gentle resuspension” of cells, which we found played an important role in maximizing efficiency. At these steps it is extremely important that cells are not directly pipetted or touched by the pipette. Rather, pipette the supernatant and resuspend cells by dispensing supernatant slowly in a clockwise motion around the top to middle edge of the tube.

Unless indicated, vortexing indicates use of benchtop vortexer at mid-high speeds (VWR Mini Vortexer, speed setting 8/10).

Please see the *Additional notes* before starting the protocol.

Protocol

1. Inoculate 5 ml of 2xYPD supplemented with adenine with a single yeast colony and grow overnight at 30 °C with shaking. We used a glass tube in a tissue culture rotator (LAB-LINE Cel-gro) at speed setting 6 (approximately 42 rpm). In parallel, incubate a 5 ml aliquot of the same growth medium as a control for contamination.

2. After 16 h, estimate cell density of the culture by transferring 100 µl from the culture to 900 µl of 2xYPD in a clean, standard cuvette, and 1000 µl of the control to a second cuvette for standardizing absorbance. Measure absorbance of 600 nm light (OD_{600}) (Ultropec 2100 Pro) and calculate OD_{600} by a) subtracting the control absorbance and b) multiplying by 10 to account for dilution factor. Reinoculate cells into 50 ml of 2xYPD supplemented with adenine, diluting to a final calculated OD of 0.2 in an Erlenmeyer flask. Grow for 3 h at 30 °C in an incubator shaker (INFORS HT Multitron Pro, 220 rpm).

3. Prepare materials for transformation as summarized in the *Additional notes*.

4. After 3 h, re-measure OD_{600} as described above except without dilution. If OD_{600} is between 0.6 and 0.7, use Table 1 to calculate the volume of culture to transfer to a sterile conical 50ml tube and proceed to Step 5. $OD_{600} > 0.7$, do not use; cancel transformation or use backup flask prepared in parallel. If $OD_{600} < 0.6$, return to incubator and re-measure after 30 minutes.

5. Spin-down culture at 2640 x *g* for 5 min. Discard supernatant and gently resuspend pellet in 10 ml sterile ddH_2O. If available, use a 2-10 ml air macropipette rather than a graduated pipette with propipetter. Spin down again at 2640 x *g* for 5 min.

6. Discard supernatant and gently resuspend pellet in 1 ml of 100 mM lithium acetate (LiAc). Transfer entire suspension to a sterile 1.5 ml microcentrifuge tube. Spin down at top speed for 10 s.

7. Discard supernatant by suction with 200 µl pipette tip. Gently resuspend cells with 100 mM LiAc to a final volume of 500 ml. Vortex cells for 1 s immediately prior to distributing 50 µl aliquots into labelled 1.5 ml microcentrifuge tubes.

8. Spin down cells at top speed for 10 s and remove supernatant by suction. Quickly vortex transformation master mix (see Additional Notes) and immediately add 326 µl to each tube containing cells.

9. To each tube, add 36 µl of DNA solution for transformation, pipetting up and down several times in supernatant. The DNA solution should contain the total desired mass of DNA for transformation, with filter-sterilized ddH_2O added up to 36 µl. A minimum of 2 ng total DNA should be used. Increasing DNA mass increases transformation efficiency.

10. Mix cells and the solution vigorously using a beadbeater (Disruptor Genie, Scientific Industries) or similar apparatus, for 1 min.

11. Heat shock cells by incubating them in a 42 ºC heat bath for 45 min.

12. Spin down cells at 6000 rpm for 2 min. Remove supernatant of all tubes by suction. Immediately add 150 µl sterile ddH_2O to each individual tube, and gently resuspend. Do not vortex.

13. After all cells have been resuspended, begin plating. This is done 5-6 plates at a time, in the following order: 1) add beads to 5-6 plates, 2) one-by-one select tubes, mix by pipetting up and down, transfer entire sample to plate (set pipette to 180µl to ensure all cells are pipetted). If transformation confers loss of an auxotrophy, pipette cells directly onto agar plates with synthetic growth medium lacking the amino acid or nucleobase of interest. Otherwise, use agar plates with YPD, and after Step 14, replica plate onto agar containing drug of interest.

14. Leave plates on bench for 30 min, then, grow in 30 ºC incubator for 2 days.

Additional Notes

We found limiting the time of cell manipulation prior to transformation increased efficiency. This is achieved by ensuring the following steps are complete before beginning Step 4:

Set a heat block to 100 ºC and a water bath to 42 ºC.

Prepare single-stranded carrier DNA from salmon sperm DNA (ssDNA) by boiling and then cooling using the following steps: a) take ssDNA aliquot (see Solutions) from -20 ºC and place directly in 100 ºC heat block for 5 min, b) remove tube and mix by inversion, c) place tube back in 100 ºC heat block for a further 5 min, then d) remove and place tube directly into ice. Cooling on ice reduces DNA re-annealing. Keep on ice until use. Only reuse boiled ssDNA once, and keep at -20 °C between uses.
1.Prepare fresh 100 mM LiAc from 1 M LiAc by diluting in sterile ddH_2O.

Label all microcentrifuge tubes.

Prepare transformation master mix of 50% PEG, 1 M LiAc and freshly prepared ssDNA. Each sample requires: 240 µl 50% PEG, 36 µl 1 M LiAc and 50 µl boiled ssDNA. Make enough for your sample number including controls, plus one.

Prepare DNA for transformation. Thaw on ice if needed. To achieve a final volume of 36 µl, dilute in filter sterilized ddH_2O if needed.

Tables:

Table 1. Determine volume of culture to use for transformation protocol (Step 5) based on measured OD

OD_{600}	Volume of culture to use (ml)
0.60	50.0
0.61	49.0
0.62	48.1
0.63	47.2
0.64	46.4
0.65	45.6
0.66	44.8
0.67	44.0
0.68	43.3
0.69	42.3
0.70	41.4

Solutions and media:

50% w/v Polyethylene glycol (PEG)

Dissolve 50 g of MW3350 PEG (Sigma, Cat. no. P3640) in a total volume of 100 ml ddH_2O and filter sterilize. To prevent evaporation, PEG should be stored in a hermetic tube. Incorrect concentration reduces transformation efficiency.

TE Buffer (pH 8.0)

10 mM Tris-HCl; 1 mM EDTA

Salmon sperm DNA (2 mg/ml, ssDNA)

Use high-molecular weight DNA sodium salt from salmon testes (Sigma, Cat. no. D1626). Dissolve 250 mg DNA in TE buffer to a final volume of 125 ml. Mix vigorously on a magnetic stirrer for 2-3 h at room temperature. Make 1 ml aliquots and store at -20 °C.

1 M lithium acetate (LiAc)

Dissolve LiAc in ddH_2O and filter sterilize. There is no need to titrate this solution, but the final pH should be between 8.4 and 8.9.

YPD growth medium

For 500 ml, combine 5g of yeast extract (Bio Basic Inc., Cat no. G0961), 10 g of bacteriological peptone (Wisent, Cat. no. 800-157-LG) and 480 ml ddH_2O in an autoclavable glass bottle. Autoclave. Prior to use, add 50 ml of sterile 20% w/v glucose solution (Fisher, Cat. no. BP350-1) and 5 ml of 100x (4.2 g/l) stock of adenine hemisulfate (Sigma, Cat. no. A9126).

2xYPD growth medium

For 500 ml, use the same recipe as YPD, except use 10 g of yeast extract and 20 g of bacteriological peptone.

YPD agar growth medium

For 500 ml, use the same recipe as YPD, except add 10 g of agar (Bioshop, Cat. no. AGR001). After autoclaving, allow to cool to 65 °C before adding glucose, adenine and drug(s). Then pour or pipette onto plates of interest and store at 4 °C after agar has solidified.

Synthetic agar growth medium lacking uracil

For 500 ml, use the same recipe as YPD agar growth medium, except replace yeast extract and bacteriological peptone with 3.35 g of yeast nitrogen base without amino acids (Wisent, Cat no. 800-152-CG) and a pre-determined mass of a prepared amino acid and nucleobase powder mix that results in a final

concentration of 90 mg/l of all 20 standard amino acids except leucine, 180 mg/l of leucine and 20 mg/l of adenine (all purchased from Sigma).

Impeders of drug selectivity in growth media

Selection for canavanine resistance must be with media lacking its non-toxic analogue arginine. Thialysine resistance selection must be with media lacking its non-toxic analogue lysine. G418/Geneticin and nourseothricin resistance selection must be with YPD medium or synthetic growth medium lacking ammonium sulfate. Synthetic growth media can be made without ammonium sulfate by replacing yeast nitrogen base with 0.85 g yeast nitrogen base without amino acids and ammonium sulfate (Wisent, Cat. no. 800-153-EG) and 0.5 g L-Glutamic acid sodium salt hydrate (Sigma, Cat. no. G1626).

Drug concentrations

200 mg/l G418 sulfate (Wisent, Cat no. 400-130-IG)

References

1. Life Technologies. Product Manual for "GeneArt® Seamless Cloning and Assembly Enzyme Mix". 2013. https://tools.thermofisher.com/content/sfs/manuals/MAN0006910_100009437_Rev02_PI_22May2013.pdf. Accessed 17 Aug 2015.

2. New England Biolabs. Gibson Assembly® Cloning Kit Instruction Manual. 2013. Subsection:"Gibson Assembly Reaction". https://www.neb.com/~/media/Catalog/All-Products/E13C03EA3FE14F12BEA0A0ECE9490093/Datacards%20or%20Manuals/ManualE5510.pdf. Accessed 17 Aug 2015.

3. Shevchuk NA, Bryksin AV, Nusinovich YA, Cabello FC, Sutherland M, Ladisch S. Construction of long DNA molecules using long PCR-based fusion of several fragments simultaneously. Nucleic Acids Res. 2004;doi:10.1093/nar/gnh014.

4. Horton RM, Hunt HD, Ho SN, Pullen JK, Pease LR. Engineering hybrid genes without the use of restriction enzymes: gene splicing by overlap extension. Gene. 1989;77:61-68.

5. Bio Basic Inc. EZ-10 Spin Column Handbook. 2011. Subsection: EZ-10 Spin Column PCR Products Purification Kit. https://store.biobasic.com/resources/productinfo/Kit%20Brochure%20Complete%20-v20.pdf. Accessed 17 Aug 2015.

6. Burke D, Dawson D, Stearns T. Methods in yeast genetics: a Cold Spring Harbor laboratory course manual. Woodbury; Cold Spring Harbor Laboratory Press; 2000. pg. 103-105.
